# Engineered bovine serum albumin-based nanoparticles with pH-sensitivity for doxorubicin delivery and controlled release

**DOI:** 10.1080/10717544.2020.1797243

**Published:** 2020-08-05

**Authors:** Zhihang Yang, Na Zhang, Teng Ma, Libo Liu, Lini Zhao, Hui Xie

**Affiliations:** a Department of Physiology, College of Basic Medicine, Shenyang Medical College, Shenyang, China; b Department of Electrical Diagnosis, Central Hospital Affiliated to Shenyang Medical College, Shenyang, China; c Department of Neurobiology, School of Life Sciences, China Medical University, Shenyang, China; d Key Laboratory of Cell Biology, Ministry of Public Health of China, and Key Laboratory of Medical Cell Biology, Ministry of Education of China, China Medical University, Shenyang, China; e Department of Pharmacology, College of Basic Medicine, Shenyang Medical College, Shenyang, China; f Department of Histology and Embryology, College of Basic Medicine, Shenyang Medical College, Shenyang, China

**Keywords:** BSA, pH-sensitivity, drug delivery, controlled release, cancer therapy

## Abstract

In this work, we prepared a stimuli-responsive system for drug delivery and controlled release by engineering the bovine serum albumin (BSA). The doxorubicin (DOX)-loaded BSA nanoparticles (NPs) were conveniently prepared using desolvation method, followed by crosslinking through Schiff base bonds, leading to pH-sensitive DOX-loaded system (DOX_s_@BSA NPs). The resulted DOX_s_@BSA NPs showed high drug loading capacity (21.4%), and the particle size was about 130 nm with narrow polydispersity and high negative surface charge (−20.5 mV). The pH-sensitivity of DOX_s_@BSA NPs was evidenced by the size changes and charge reversal after incubation at different pH values. The DOX_s_@BSA NPs showed high serum stability which indicated the prolonged circulation time. The *in vitro* drug release experiment showed that the release of DOX was obviously accelerated by acidity because of disassembly of NPs induced by cleavage of Schiff base bonds. The drug release mechanism was thoroughly studied using a semi-empirical model, further confirming the pH played an important role in drug controlled release process. The results of cytotoxicity assay revealed that DOX_s_@BSA NPs exhibited much higher toxic effects for tumor cells in comparison to the free DOX control. Collectively, these results demonstrated that DOX_s_@BSA NPs might be potential application for drug delivery and controlled release in cancer chemotherapy. Moreover, this work also showed that preparation of stimuli-responsive drug delivery system by engineering the commercial biomaterials could be a promising method to develop multi-functional nanomedicine.

## 1. Introduction

Cancer has become the leading killer of humans, causing 9.6 million of death and US$1.16 trillion of economic cost every year (Copur 2019; Datta et al. 2019; Ferlay et al. 2019). With the rapid development of nanotechnology and material engineering, some emerging therapies including photothermal therapy (PTT), photodynamic therapy (PDT), gene therapy and immunotherapy, have been developed in these years (Mellman et al. 2011; Zhang et al. 2017; Zeng et al. 2019; Riley et al. 2019; Xu et al. 2020). However, the therapeutic efficacy and biosafety have to be further improved and evaluated before clinic use (Yang et al. 2013; Dickmann et al. 2014; Fakhoury 2016). So far, traditional chemotherapy is still the most common and useful approach to treat the cancer clinically. In these decades, many chemical anticancer drugs have been synthesized and prepared, such as doxorubicin (DOX), paclitaxel (PTX), and camptothecin (CPT), for cancer chemotherapy (Gordon et al. 1995; Lai et al. 2007; Min et al. 2008; Oh et al. 2010). However, the small molecular drugs have their own limitations in clinical applications, such as quick clearance by reticuloendothelial system (RES), low anticancer efficacy and severe side-effect (Brannon-Peppas and Blanchette 2004; Guo and Huang 2011; Sandhu et al. 2015). For example, DOX which can interact with DNA by intercalation and inhibition of macromolecular biosynthesis is one of the most effective drugs for a wide range of cancers including breast cancer, bladder cancer, lymphoma, and acute lymphocytic leukemia (Soma et al. 2000; Sharma 2017; Sun et al. 2019; Zhou et al. 2020). Even though DOX was approved for medical use in the United States 45 years ago (Agarwal et al. 2011), the side-effects severely limit the wide use in clinic, such as hair loss, bone marrow suppression, vomiting, rash, and inflammation of the mouth, especially the dilated cardiomyopathy which can lead to congestive heart failure (Singal and Iliskovic 1998; Chatterjee et al. 2010; Dong et al. 2017). To overcome these obstacles, drug delivery system (DDS) has been thoroughly investigated and extensively used to develop nano-scale formulations for improving the therapeutic efficacy and reducing the side-effects, especially the stimuli-responsive biomaterials-based DDSs (Gong et al. 2012; Mura et al. 2013; Yin et al. 2013; Li et al. 2014; Sun et al. 2014; Masood 2016; Qiao et al. 2019). Considering the specific tumor microenvironments such as low pH (Zhang et al. 2019), high glutathione (GSH) concentration and special enzyme (Kong et al. 2016; Li et al. 2019), various stimuli-responsive biomaterials including biopolymer (Li et al. 2018), inorganic nanoparticles (Baeza et al. 2015), and metal-organic frameworks have been developed and used as chemical anticancer drug carriers to improve the cancer chemotherapy (Chen et al. 2018; Wang et al. 2020). However, before these multi-functional nanocariers can be considered for clinical applicability, two vital challenges have to be addressed. One is the synthesis of these biomaterials should need to be adapted for scaling-up, so as to minimize polydispersity in size and surface chemistry. The other one is the biosafety including cytotoxicity, biocompatibility and biodegradation which have to be further evaluated. Conversely, some traditional and commercial biomaterials-based NPs (e.g. poly(lactic acid) (PLA), poly lactic-*co*-glycolic acid (PLGA)) used as drug delivery systems do not exhibit multi-functionality, such as pH-triggered drug release behavior, charge reversal, improved cellular uptake (Byrne and Deasy 2002; Makadia and Siegel 2011; Larrañeta et al. 2016; Saini et al. 2016). Particularly, although the commercial BSA shows nontoxic effect and high biosafety (Mariam et al. 2016; Saha et al. 2019), BSA does not have the stimuli-responsiveness for controlled release of encapsulated cargos. It’s of great interest and significance to develop functional drug delivery system based on BSA with high drug loading capacity and stimuli-responsive property.

In this work, we designed and developed an engineered BSA-based NP for delivery of DOX with pH-triggered release profile. Anticancer drug DOX is physically loaded into the BSA-based NPs using desolvation method, resulting in DOX-loaded BSA NPs (named after DOX@BSA NPs). And then, glutaraldehyde is used to crosslink the BSA through the interaction between aldehyde and amine residues, resulting in Schiff base bond which is pH-sensitive (named after DOX_s_@BSA NPs) (Feng et al. 2017; Peng et al. 2019; Xu et al. 2019). The physicochemical properties of DOX_s_@BSA NPs including particle size, zeta-potential, serum stability, drug release behavior and mechanism, and cytotoxicity were investigated by a variety of experimental techniques.

## 2. Materials & methods

### 2.1. Materials

Doxorubicin hydrochloride (DOX-HCl) was purchased from Wuhan Yuan Cheng Gong Chuang Co. Bovine serum albumin (BSA, ≥98%), glutaric acid (≥99%), glutaraldehyde, triethylamine (TEA, ≥99%) and methylthiazoltetrazolium (MTT) were purchased from Sigma (St. Louis, MO). Dulbecco’s modified eagle media (DMEM) growth media, fetal bovine serum (FBS), trypsin, penicillin and streptomycin, were all purchased from Invitrogen; NIH 3T3, A549, A2780 and NCL-H460 cell lines were obtained from the American Type Culture Collection (ATCC). All other reagents were used as received.

### 2.2. Preparation of DOX_s_@BSA NPs

BSA NPs were prepared by the desolvation technique (Chen et al. 2014; Chu et al. 2015). Briefly, BSA was first dissolved in deionized water at the concentration of 20 mg/mL with stirring at room temperature. The dimethyl sulfoxide (DMSO) was then added into the BSA solution (1: 5, v/v) with stirring. Thirty minutes later, ethanol was quickly added into the BSA solution (3.5: 1, v/v) under low stirring at room temperature. The reaction was carried on for 2 h. To prepare the stable and pH-responsive BSA NPs, glutaraldehyde (2%, 120 μL per 1 mL BSA solution) was added, and the reaction was carried on for 24 h. After that, the resulted solution was centrifuged at 20,000 g for 30 min at 4 °C. The received pellets were re-suspended in deionized water, and centrifuged again. This step was repeated three times to remove organic solvents. The obtained crosslinked BSA NPs (sBSA NPs) was re-suspended in phosphate buffer solution (PBS, pH 7.4) for the follow-up experiments. Furthermore, glutaric acid was used to replace the glutaraldehyde to prepare pH-insensitive BSA NPs (iBSA NPs) which was used as negative control in future.

To prepare the pH-responsive DOX-loaded BSA NPs (DOX_s_@BSA NPs), the DOX-HCl (5 mg, 10 mg, 20 mg) was dissolved into DMSO, and then the TEA (0.1 μL of TEA per 1 mg of DOX) was added with stirring. The mixed solution was stirred at least for 30 min in dark at room temperature. BSA solution (1 mL, 20 mg/mL) was added and incubated for 15 min. The followed steps were similar. After desolvation and crosslink, the obtained solution was centrifuged (20 000 rpm, 30 min) to remove the organic solvents and the unencapsulated drugs. After that, the DOX_s_@BSA NPs were obtained and kept in 4 °C for use in future.

### 2.3. Characterization of DOX_s_@BSA NPs

The particle size, size distribution (polydispersity index, PDI) and zeta-potential of iBSA NPs, sBSA NPs and DOX_s_@BSA NPs were measured by dynamic light scattering (DLS, Malvern Zetasizer Nano S, Malvern, UK).

To evaluate the stability of DOX_s_@BSA NPs, 1 mL of DOX_s_@BSA NPs (1 mg/mL) was re-suspended into 1 mL of PBS at pH 7.4 with 20% FBS, followed by incubation for different time at 37 °C. The particle size of sample was recorded every day. Furthermore, DOX_s_@BSA NPs were re-suspended into PBS (pH 7.4) or 5% glucose at the concentration of 1 mg/mL. And then, the solution was diluted at 1/1, 1/10, 1/100 and 1/1000 of original concentration. The particle size of sample was then measured using DLS.

To study the pH-sensitivity, the particle size and zeta-potential of iBSA NPs, sBSA NPs and DOX_s_@BSA NPs in PBS at different pH values were measured. In brief, the NPs were re-suspended into PBS with different pH values (3.0, 4.0, 5.0, 5.5, 6.5, 7.4 and 8.0) at the concentration of 1 mg/mL, followed by incubation for 24 h at 37 °C. And then, the samples were measured using DLS aforementioned.

### 2.4. Drug loading capacity

UV-vis spectrophotometer (UV-2450, Shimadzu, Japan) was used to confirm the drug loading content (LC) and encapsulated efficiency (EE) of DOX_s_@BSA NPs. Briefly, 1 mL of supernate after centrifugation was measured using UV-vis spectrophotometer at 480 nm to confirm the concentration of DOX. And then, the amount of unloaded DOX was calculated according to the standards curve. The LC was defined as mass ratio of (DOX in feed-unloaded DOX) to DOX_s_@BSA NPs. The EE was defined as mass ratio of (DOX in feed-unloaded DOX) to DOX in feed.

### 2.5. In vitro DOX release from DOX_s_@BSA NPs

The *in vitro* DOX release profile from DOX_s_@BSA NPs was studied using dialysis method. This experiment was performed at a quite low drug concentration to mimic the sink condition (Zhang et al. 2012). In brief, 4 mg of DOX_s_@BSA NPs was re-suspended into 4 mL of PBS at pH 7.4, 6.5 and 5.0, and transferred into a dialysis bag (molecule weight cutoff, MWCO, 3500 Da). This dialysis bag was immersed into the corresponding PBS (46 mL, pH 7.4, 6.5 or 5.0) in a beaker at 37 °C with stirring at 110 rpm. At predetermined time interval, 1 mL of solution in the beaker was taken out for UV-vis analysis, and 1 mL fresh PBS was added. The free DOX was used as control. The cumulative drug release percent (*E*_r_) was calculated according to the following equation:

$$E_{r}(\%)\frac{V_{e}\sum_{1}^{n-1}C_{i}+V_{0}C_{n}}{m_{\mathrm{DOX}}}\times 100\%$$

where, *m*_DOX_ was the loaded DOX in BSA NPs, *V*_e_ and *V*_0_ were, respectively, the volume of buffer in dialysis bag (4 mL) and the total volume of buffer (50 mL), and *C*_i_ was the DOX concentration in the *i*_th_ sample.

### 2.6. Cell culture

The NIH 3T3, A549, A2780 and NCL-H460 cell lines were cultured according to the standard protocol from supplier with a few modifications. In brief, the cells were cultured in fresh DMEM containing 10% (v/v) FBS, 100 units/mL penicillin, and 100 µg/mL streptomycin in a flask, and incubated at 37 °C in a CO_2_ (5%) incubator. The cells were checked every day, and approximately separated every 2 or 3 days.

### 2.7. Cytotoxicity test

The cytotoxicity of BSA NPs, free DOX and DOX_s_@BSA NPs against NIH 3T3, A549, A2780 and NCL-H460 cells were investigated by the standard MTT assay (Wei et al. 2017). As a typical experiment, cells were seeded into 96-cell plate at density of 1 × 10^4^ cells per well in 200 µL of DMEM. The plate was incubated for 24 h. After that, the medium was removed, and pre-prepared samples in DMEM were added into the well. For BSA/DMEM solution, the series of concentrations were 5.0, 10, 100, 200, 300 and 400 µg/mL. For free DOX/DMEM and DOX_s_@BSA NPs/DMEM, the concentrations of DOX were 0.1, 0.5, 1.0, 2.0, 5.0, 10 and 20 µg/mL. Pure DMEM was used as background, and cell with medium was used as control. After 48 h incubation, the medium was removed and the well was washed three times using PBS. 100 µL of MTT solution (1 mg/mL in DMEM) was added into each well. And the plate was further incubated for 4 h. And then, the solution was removed, and 100 µL of DMSO was added to dissolve the crystal. After shaking for 15 min, the plate was measured at by a microplate reader (Multiskan Spectrum, Thermo Scientific, Finland) at 570 nm. The cell viability was calculated using the following equation:

Cell    viability=A−sampleAblankA−controlAblank  ×  100  %

where, *A* was the absorbance of control, blank and sample at 570 nm. The cytotoxicity test was performed in replicates of six wells.

### 2.8. Statistical analysis

All data were expressed as the mean ± standard deviation (S.D.). Statistical analysis was conducted using paired Students’s *t*-test analysis.

## 3. Results & discussion

### 3.1. Preparation and characterization of DOX@BSA NPs

The pH-sensitive DOX-loaded BSA NPs were prepared using desolvation technique, followed by crosslinking using glutaraldehyde through Schiff base bonds. The pH-insensitive BSA NPs (iBSA NPs) were also prepared as a negative control. As shown in Figure 2(A), iBSA NPs and sBSA NPs were characterized by dynamic light scattering (DLS), showing that their particle sizes were similar and approximately 120 nm in diameter. After loading DOX, the particle size of DOXs@BSA NPs slightly increased (*c.a.* 130 nm) compared with sBSA NPs, resulting from the encapsulation of DOX molecules in the core of NPs through the hydrophobic interaction (Xu et al. 2019). The similar results could be found in Figure 2(B). Furthermore, the narrow distribution curve of BSA-based NPs revealed the low nanoparticle polydispersity and high uniformity, demonstrating that desolvation and crosslink could be useful methods to prepare multi-functional BSA NPs. The TEM image of DOXs@BSA NPs was showed in supplementary Figure S1. The particle size was approximately 120 nm which was slightly less than that confirmed by DLS. The reason could be due to shrinking of the particles during the drying process prior to TEM analysis. In addition, the DLS data presented an intensity average, which could also be another reason for the discrepancy between DLS and TEM data. Figure 2(C) showed the zeta-potential of iBSA NPs, sBSA NPs and DOXs@BSA NPs. For the three BSA-based NPs, the zeta-potential was negative, indicating that these BSA-based NPs were able to avoid the interaction with the most proteins with negative charge in biological circulation. In addition, the zeta-potential of DOXs@BSA NPs was slightly higher compared with those of iBSA NPs and sBSA NPs, resulting from the loading of positively charged DOX molecules.

To further confirm the optimal DOX formulation, different mass ratios of drug to BSA were performed and studied. The particle size, PDI, LC and EE were shown in Table 1. When the mass ratio was 1: 4 (5 mg of DOX and 20 mg of BSA), the LC and EE were 15.6% and 71.8%, respectively. With increase of mass ratio to 1: 2 (10 mg of DOX and 20 mg of BSA), the LC was obviously increased to 21.4%, while the EE was slightly decreased to 62.0%. When the mass ratios was 1: 1 (20 mg of DOX and 20 mg of BSA), the LC (25.2%) was slightly increased compared with that at mass ratio of 1: 2, but the EE was markedly decreased to 47.9%. In addition, the particle sizes of DOX_s_@BSA NPs at different mass ratios were about 130 nm which was slight higher than that of blank sBSA NPs. The PDI was increased after loading of DOX molecules in BSA NPs due to the increased core. The PDI at mass ratio 1: 1 was the highest, possibly resulting from the aggregation of excessive unloaded DOX molecules. Collectively, the formulation at mass ratio of 1: 2 was the optimal, and would be used in the followed studies.

**Table 1.** Particle size, polydispersity index (PDI), drug loading content (LC) and encapsulation efficacy (EE) of DOX_s_@BSA NPs at different mass ratios of drug and BSA carriers.

Carrier (20 mg)	DOX (mg)	LC (%)^a^	EE (%)^a^	Size (nm)^b^	PDI^b^
BSA	0	–	–	120.3	0.138
	5	15.6	71.8	130.1	0.143
	10	21.4	62.0	132.2	0.145
	20	25.2	47.9	135.7	0.156

^a^Measured by UV-vis, ^b^measured by DLS.

### 3.2. Ph-sensitivity of DOX_s_@BSA NPs

Next, to study the pH-sensitivity of DOX_s_@BSA NPs, the particle size and zeta-potential of BSA-based NPs after incubation in PBS with different pH values for 24 h were recorded, as shown in Figure 3(A,B). The particle size of iBSA NPs at pH 7.4 was 121.6 nm. With the decrease of pH, the particle size was dramatically increased (198.1 nm at pH 5.0 and 217.5 nm at pH 3.0) because of swelling of iBSA NPs. For pH-sensitive sBSA NPs, the particle size was firstly increased with decrease of pH values, and the trend was similar to that of iBSA NPs due to the swelling of NPs. In contrast, when the pH was less than 5.0, the particle size of sBSA NPs was decreased to about 15 nm. The reason was that the sBSA NPs were disassembled into BSA, resulting from the cleavage of Schiff base bonds triggered by acidity. The same trend could be observed for DOX_s_@BSA NPs at different pH conditions. Figure 3(B) showed the surface charge of iBSA NPs, sBSA NPs and DOX_s_@BSA NPs at different pH values. The zeta-potential of three BSA-based NPs was significantly increased with the decrease of pH value. When the pH decreased from 8.0 to 3.0, the surface charge was reversed from negative to positive indicated by the zeta-potential characterization, attributing to the protonation of amine residues in BSA under acidic conditions. Additionally, DOX_s_@BSA NPs showed slightly higher zeta-potential in comparison to that of blank BSA NPs, because the amine residues of DOX might also be ionized and enhance the surface charge. In summary, the changes in particle size and surface charge of BSA-based NPs showed that the pH-sensitivity. Then, the NPs system which can be used in biomedical application should have high stability. Herein, we investigate the serum stability of DOX_s_@BSA NPs *in vitro*. The particle size of DOX_s_@BSA NPs after incubation in PBS at pH 7.4 with 20% FBS at 37 °C for different time was measured, as shown in Figure 3(C). The particle size of DOX_s_@BSA NPs was increased from 132.2 nm to 146. 1 nm after incubation for 5 days, but the increasing range was less than 20%, suggesting the high serum stability. The similar results could be found for the zeta-potential changes of DOX_s_@BSA NPs (supplementary Figure S2). Moreover, the particle size of DOX_s_@BSA NPs in PBS (pH 7.4) or 5% glucose solution after dilution showed no significant changes (Figure 3(D)), indicating the high stability of DOX_s_@BSA NPs. These findings demonstrated that the DOX_s_@BSA NPs may have prolonged circulation time which is the precondition for drug delivery.

### 3.3. *Dox release profile* in vitro

Next, to confirm that DOX molecules could be released from DOX_s_@BSA NPs dependent on pH, the drug release behavior *in vitro* of DOX_s_@BSA NPs was performed in PBS at different pH values at 37 °C. The different pH conditions were selected to simulate the normal physiological condition (7.4) and tumor microenvironment (pH 6.5 and 5.0). The *in vitro* drug release profiles of DOX_s_@BSA NPs at pH 7.4, 6.5 and 5.0 were shown in Figure 4. At pH 7.4 and 5.0, the DOX release rate of free DOX was rapid, and the cumulative release amount of DOX was higher than 90% for 12 h at pH 7.4 or 5.0. Compared with free DOX, DOX_s_@BSA NPs exhibited the sustained drug release behaviors. At pH 7.4, the DOX release rate was slow, and the cumulative release amount of DOX from DOX_s_@BSA NPs was only 29.7% and 38.6% for 24 h and 168 h, respectively. In contrast, when the pH decreased to acidic condition, the DOX release rate was obviously accelerated, and the cumulative release amount was higher than 55% (pH 6.5) and 70% (pH 5.0) for 24 h, respectively. Furthermore, for 148 h, the cumulative release amount was higher than 75% (pH 6.5) and 85% (pH 5.0), respectively. Furthermore, the DOX release rates of DOX-loaded iBSA NPs at pH 7.4 and 5.0 were much lower compared with those of DOX_s_@BSA NPs (supplementary Figure S3). These results demonstrated that the acidity can significantly facilitated the drug release rate from DOX_s_@BSA NPs, possibly resulting from the disassembly of NPs induced by the pH-triggered cleavage of Schiff base bonds under acidic condition. Furthermore, DOX molecules become more hydrophilic and water-soluble due to the protonation of amine residues in DOX. In summary, the pH-triggered drug release profiles indicated that the DOX_s_@BSA NPs might be used for drug delivery and controlled release for cancer chemotherapy.

### 3.4. Dox release mechanism

We next study the DOX release mechanism of DOXs@BSA NPs. Although the drug release mechanism of drug-loaded NPs was incompletely understood until now, a comprehensive semi-empirical model has been established to investigate this complex mechanism (Siepmann and Peppas 2012), as follows:

$$\text{log}\;\left(\frac{M_{t}}{M_{\infty }}\right)=n\;\text{log}\;t+\;\text{log}\;k$$

where, *M*_t_/*M*_∞_ was the cumulative drug release amount at time *t*. *k* was a constant which was related to the drug release rate. *n* was the release exponent which indicated the type of drug release mechanism. When *n* was about 0.43, the release mechanism corresponded to Fickian diffusion. When *n* was less than 0.43, the release mechanism might be combination of diffusion and erosion control. When *n* was higher than 0.43 but lower than 0.85, it could be anomalous transport mechanism. When *n* was 0.85, the mechanism could be swelling-controlled mechanism.

Basing on this, we further analyzed the DOX release mechanism from DOXs@BSA NPs at pH 7.4, 6.5 and 5.0. The release process was divided into two stages (0–24 h and 24–168 h), followed by analyzing using the model. Figure 5 showed the theoretical fitted curves based on the experimental data, and the fitting paramenters (*n* and *k*) at pH 7.4, 6.5 and 5.0 were shown in Table 2. Good linearity could be observed for two stages of drug release profiles at pH 7.4, 6.5 and 5.0 (Figure 5). In the case of pH 7.4, the *n* value at the first stage was 0.425 (≈ 0.43), indicating that the DOX release mechanism from DOXs@BSA NPs was Fickian diffusion. The reason could be that the DOXs@BSA NPs had high stability and maintain compact in PBS at pH 7.4, and the DOX molecules could only diffuse from the NPs. With the increase of time, the *n* value at the second stage was 0.122 which was much less than 0.43, suggesting that the DOX release mechanism corresponded to combination of diffusion and erosion control because of swelling and erosion of BSA NPs. When the pH decreased to acidic condition, the *n* value at the first stage was 0.505 (pH 6.5) and 0.538 (pH 5.0) (0.43 < *n* < 0.85) which revealed that the DOX release behavior accorded with the anomalous transport mechanism, resulting from the disassembly of DOXs@BSA NPs induced by cleavage of Schiff base bonds (Lv et al. 2014). At the second stage, the *n* values (0.111 for pH 6.5 and 0.090 for 5.0) were much lower than 0. 43, demonstrating that the DOX release mechanism was combination of diffusion and erosion control. In addition, the *n* value at pH 6.5 or 5.0 was much higher than that at pH 7.4, showing that the release rate of DOX was accelerated under acidic condition. Taken together, the pH value played a central role in DOX release from DOXs@BSA NPs. The release rate and cumulative release amount could be enhanced by the decrease of pH from 7.4 to 5.0, revealing the DOXs@BSA NPs had pH-triggered drug release profile and might be used for drug delivery and controlled release in biomedical applications.

**Table 2.** Release exponent (*n*) and rate constant (*k*) of DOX from DOXs@BSA NPs in PBS at pH 7.4, 6.5 or 5.0 at 37 °C.

DOXs@BSA NPs	pH	*n* (0–24 h)	*k* (0–24 h)	*n* (24–168 h)	*k* (24–168 h)
	7.4	0.425	0.086	0.122	0.203
DOX	6.5	0.505	0.125	0.111	0.427
	5.0	0.538	0.151	0.090	0.544

### 3.5. Cytotoxicity

Since we have demonstrated that DOXs@BSA NPs might be used for drug delivery and controlled release, we next evaluated the cytotoxicity against normal cells and tumor cells *in vitro*. MTT assay was performed to study the cytotoxicity of BSA and DOXs@BSA NPs for NIT 3T3 cells (Figure 6(A)) and tumor cell lines A549 (Figure 6(B)), A2780 (Figure 6(C)) and NCL-H460 (Figure 6(D)), respectively. As shown in Figure 6(A), even at the highest concentration (400 µg/mL), the cell viability was still higher than 90%. This result displayed that no toxic effect of BSA for NIH 3T3 cells. High cytotoxicity of DOXs@BSA NPs was observed for the three tumor cell lines. For A549 cells, the cell viability was, respectively, 17.6% and 8.9% for free DOX and DOXs@BSA NPs at the drug concentration of 20 µg/mL. And the IC50 values were 1.72 µg/mL and 0.85 µg/mL for free DOX and DOXs@BSA NPs, respectively. For A2780 cells, IC50 values for free DOX and DOXs@BSA NPs were 4.90 µg/mL and 1.87 µg/mL, respectively, and the DOXs@BSA NPs showed much higher toxic effect against A2780 cells compared with free DOX. For NCL-H460 cells, the cell viability was 14.2% and 9.3% for free DOX and DOXs@BSA NPs at the highest drug concentration, respectively. The IC50 values for free DOX and DOXs@BSA NPs were 0.97 µg/mL and 0.65 µg/mL, respectively. These results demonstrated that the DOXs@BSA NPs exhibited much higher cytotoxicity for tumor cells in comparison to free DOX formulation. The reason might be that the tumor cells could show multidrug resistance for free DOX through the P-gp pumping effect (Alakhov et al. 1999; Qiu et al. 2014). However, the DOXs@BSA NPs could be internalized *via* endocytosis by the tumor cells, and then escaped from the endo/lysosomes, followed by releasing the DOX molecules to induce the apoptosis of tumor cells (Zhang et al. 2009; Oh and Park 2014). Therefore, the DOXs@BSA NPs could inhibit the growth of tumor cells more effectively compared with free DOX. Here, these findings revealed that the DOXs@BSA NPs exhibited much higher cytotoxicity for tumor cells compared with the control free DOX, further indicating the potential application in cancer chemotherapy.

## 4. Conclusion

**Figure 1.**
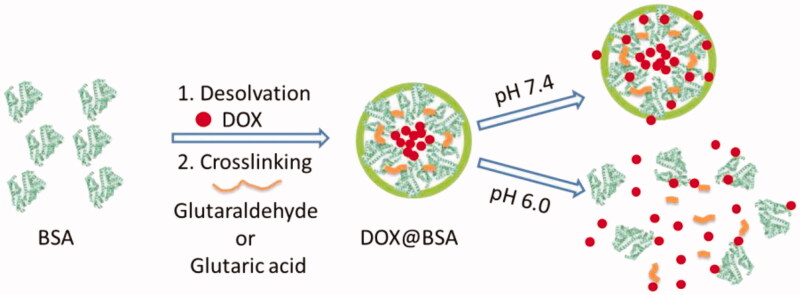
Schematic illustration of fabrication of pH-responsive DOX_s_@BSA NPs with pH-triggered drug release profile.

**Figure 2.**
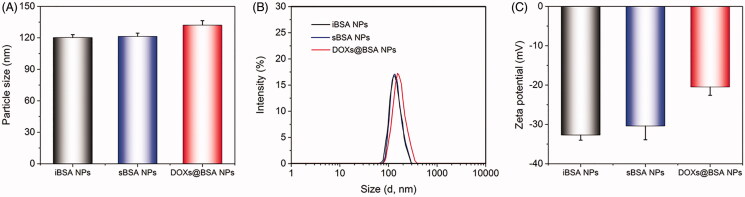
Characterization of BSA-based NPs at pH 7.4 in PBS at room temperature. Particle size (A), distribution (B) and zeta-potential (C) of iBSA NPs, sBSA NPs and DOX_s_@BSA NPs measured by DLS (*n* = 3, mean ± SD).

**Figure 3.**
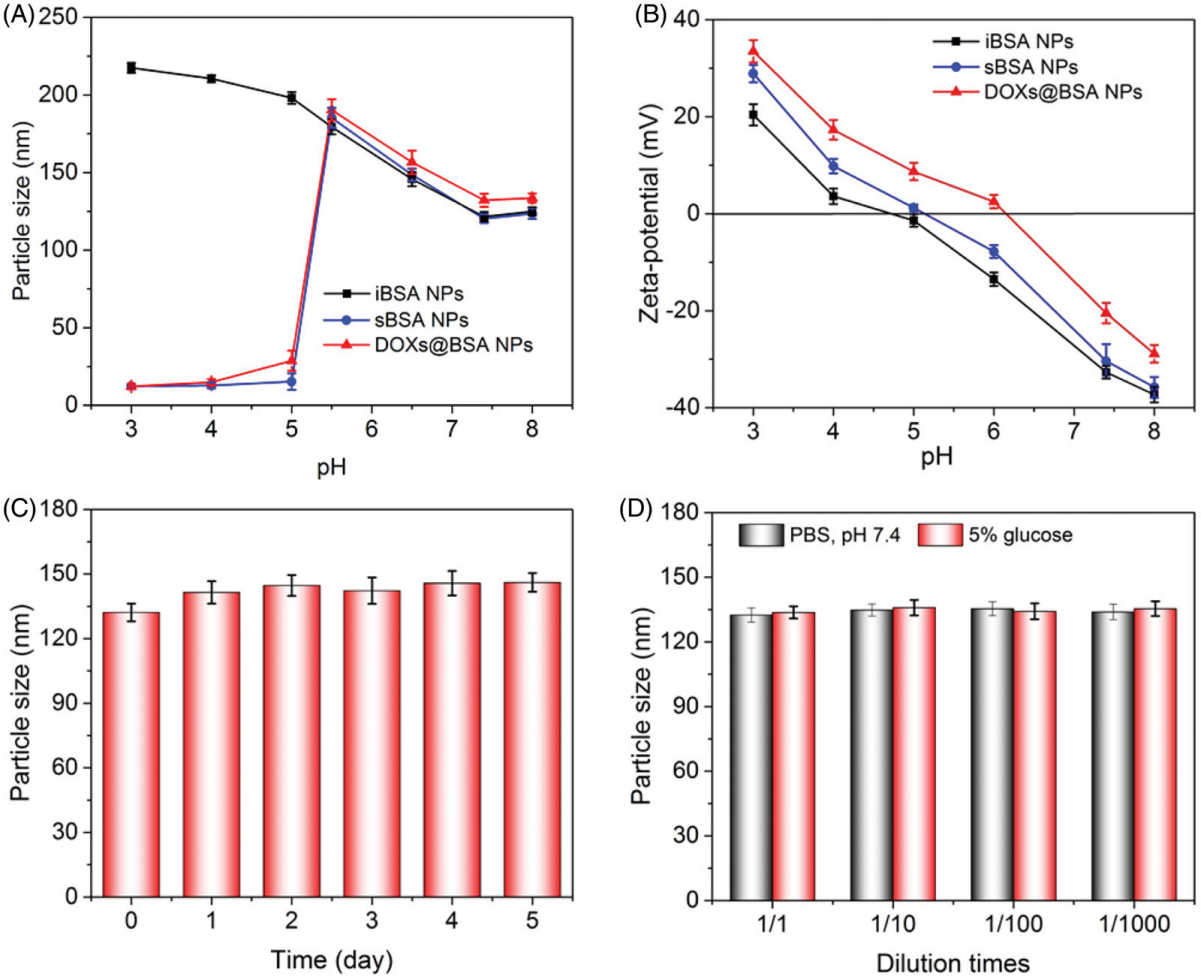
Particle size (A) and zeta-potential (B) of DOX-based NPs incubated in PBS at different pH values for 24 h. (C) Particle size of DOX_s_@BSA NPs in PBS at pH 7.4 in the presence of 20% FBS after incubation for 5 days at 37 °C. (D) Particle sizes of the DOXs@BSA NPs in 5% glucose and PBS, pH 7.4 upon dilution at 1/1, 1/10, 1/100 and 1/1000 of the original concentration of the NPs, which was 2 mg/mL (*n* = 3, mean ± SD).

**Figure 4.**
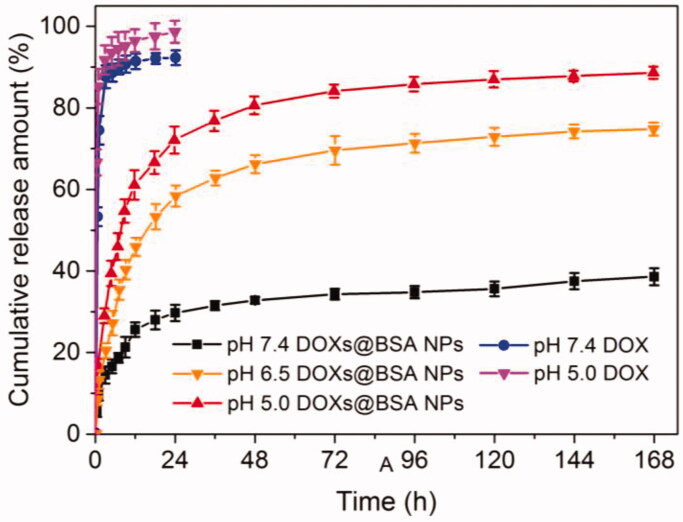
The *in vitro* release profiles of DOX from DOXs@BSA NPs in different PBS (pH 7.4, 6.5 and 5.0) solutions. The release profiles of free DOX at pH 7.4 and 5.0 were investigated and used as control (*n* = 3, mean ± SD).

**Figure 5.**
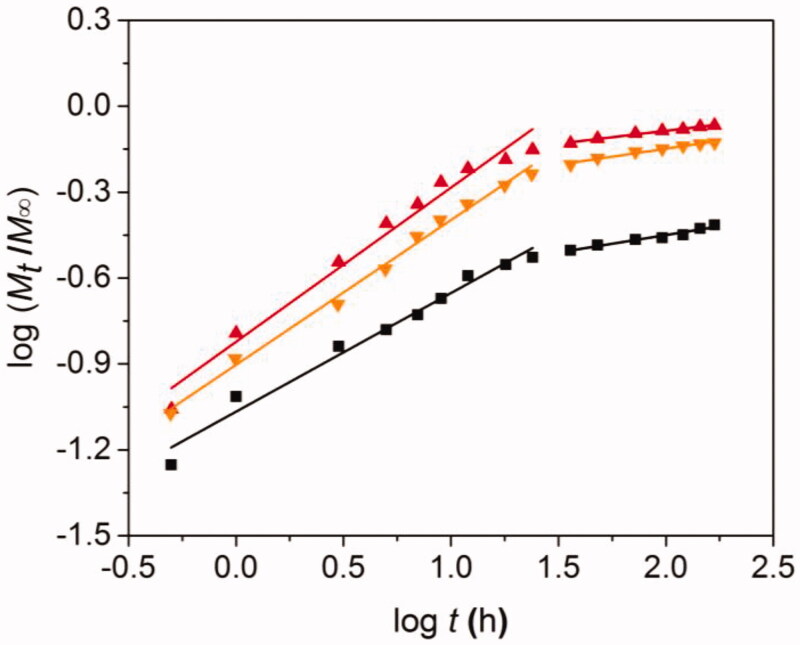
Plots of log (*M*_t_/*M*_∞_) against log *t* for DOX release from DOXs@BSA NPs at pH 7.4, 6.5 and 5.0.

**Figure 6.**
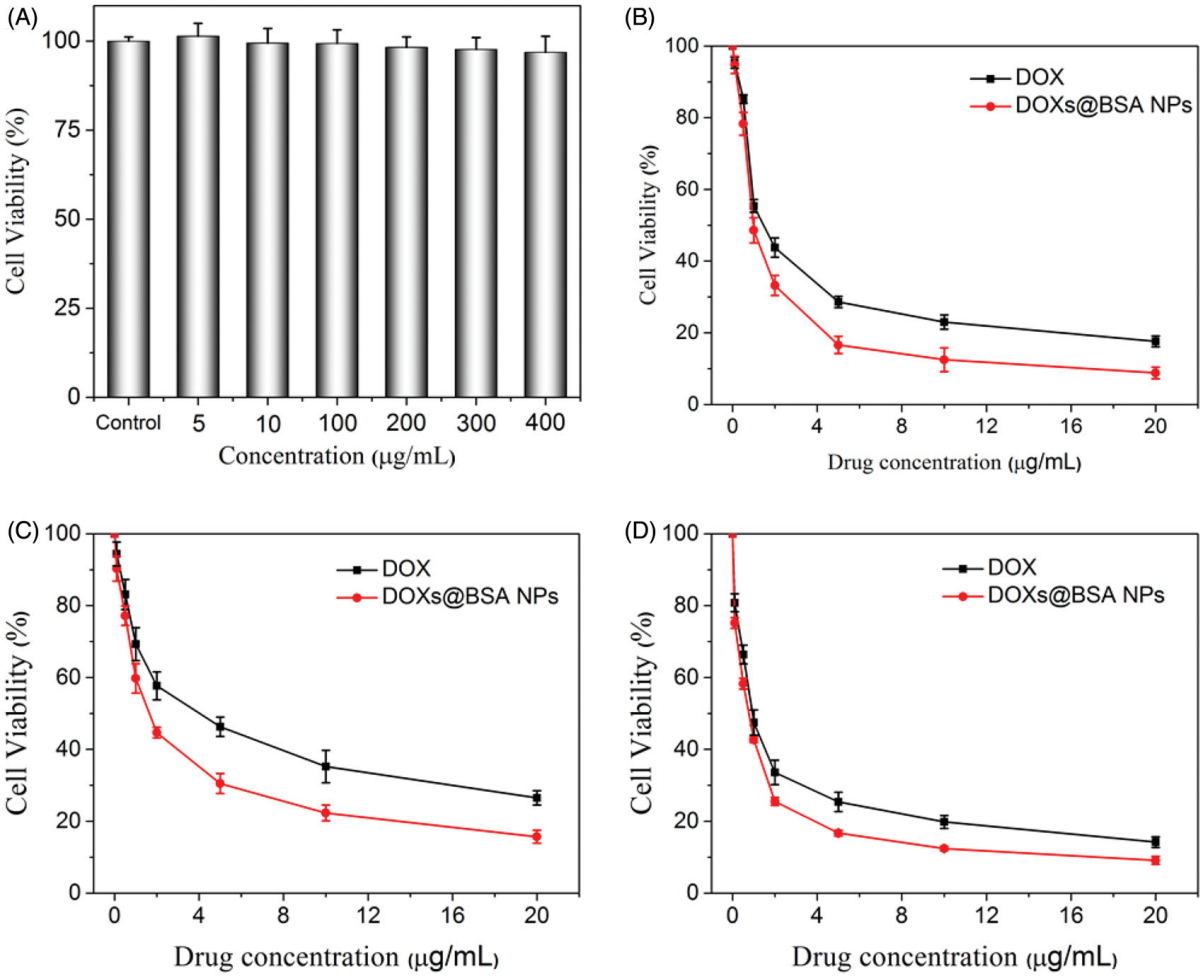
(A) Cell viability of NIH 3T3 cells treated with blank sBSA NPs for 48 h in concentration specified. Cell viability of A549 (B), A2780 (C), and NCL-H460 (D) cells treated with free DOX and DOXs@BSA NPs for 48 h in concentration specified (*n* = 6, mean ± SD).

In this work, we reported a BSA-based drug delivery system with pH-triggered drug release profile for delivery and controlled release of DOX. The DOX-loaded BSA NPs was prepared using desolvation method, followed by crosslinking through pH-sensitive Schiff base bonds, resulting in pH-responsive DOX_s_@BSA NPs for drug delivery and controlled release (Figure 1). The engineered BSA NPs were able to efficiently load DOX with high drug loading content and encapsulation efficacy (Table 1). The particle size of DOX_s_@BSA NPs was about 130 nm, and the surface charge was negative (Figure 2). DOX_s_@BSA NPs showed high serum stability and pH-sensitivity, as indicated by size changes and drug release profiles under different conditions (Figures 3 and 4). We further investigated the drug release mechanism under normal physiological condition (pH 7.4) and tumor microenvironment condition (pH 5.0) using semi-empirical equation (Figure 5). Next, we evaluated the toxic effect of BSA for NIH 3T3 cell, and the cytotoxicity of DOX_s_@BSA NPs for three tumor cell lines. The results showed that DOX_s_@BSA NPs had much higher cytotoxicity compared with free DOX (Figure 6). This study not only reported a pH-responsive nanomedicine which might be used in cancer therapy, but also showed that preparation of stimuli-responsive DDS by engineering the commercial biomaterials could be a promising method to develop multi-functional way.

## Supplementary Material

Supplemental Material
