# Supplementary material for: Engineered bovine serum albumin-based nanoparticles with pH-sensitivity for doxorubicin delivery and controlled release
Source: Drug Deliv. 2020 Aug 5;27(1):1156–64. doi: 10.1080/10717544.2020.1797243 (PMC7470134; doi:10.1080/10717544.2020.1797243)
Supplement: Supplemental Material [file IDRD_A_1797243_SM0260.docx]

**Supporting Information**

**Engineered bovine serum albumin-based nanoparticles with pH-sensitivity for doxorubicin delivery and controlled release**

Zhihang Yang^1,*^, Na Zhang^2^, Teng Ma^3,4^, Libo Liu^3,4^, Lini Zhao^5^, and Hui Xie^6^

^1^Department of Physiology, College of Basic Medicine, Shenyang Medical College, Shenyang 110034, China

^2^Department of Electrical Diagnosis, Central Hospital Affiliated to Shenyang Medical College, Shenyang 110024, China

^3^Department of Neurobiology, School of Life Sciences, China Medical University, Shenyang, China

^4^Key Laboratory of Cell Biology, Ministry of Public Health of China, and Key Laboratory of Medical Cell Biology, Ministry of Education of China, China Medical University, Shenyang 110122, China

^5^Department of Pharmacology, College of Basic Medicine, Shenyang Medical College, Shenyang 110034, China

^6^Department of Histology and Embryology, College of Basic Medicine, Shenyang Medical College, Shenyang 110034, China


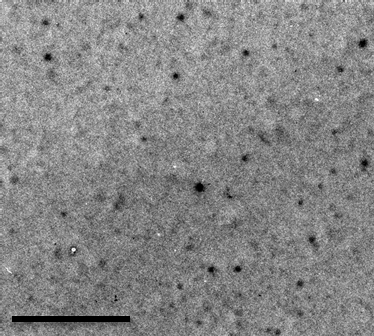


Figure S1. The TEM image of DOXs@BSA NPs in PBS at pH 7.4. Scale bar 1 µm.


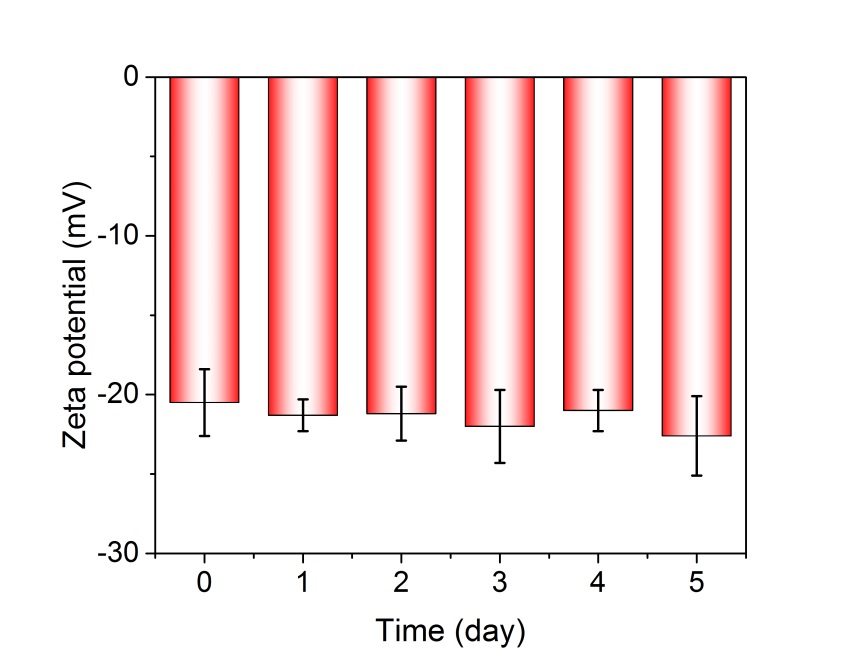


Figure S2. Zeta-potential of DOXs@BSA NPs in PBS at pH 7.4 after incubation for different time at 37 °C.


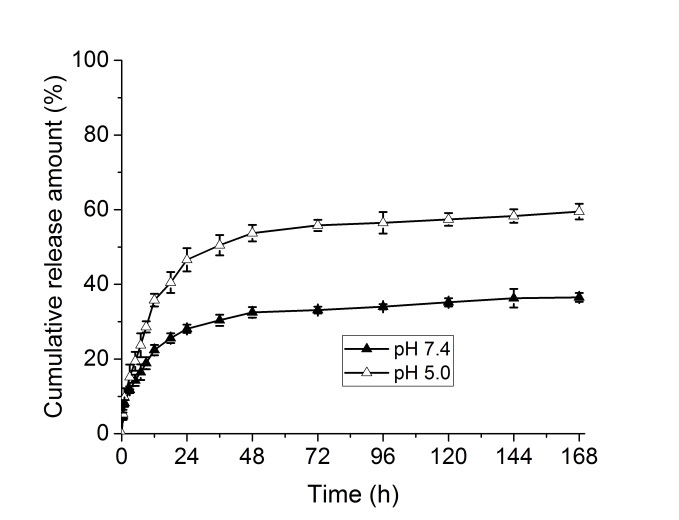


Figure S3. The *in vitro* release profiles of DOX from DOX-loaded iBSA NPs in different PBS (pH 7.4 and 5.0) solutions. n = 3, mean ± SD.
